# Association between the methylenetetrahydrofolate reductase C677T polymorphism and hepatocellular carcinoma risk: a meta-analysis

**DOI:** 10.1186/1746-1596-4-39

**Published:** 2009-11-24

**Authors:** Fei Jin, Li-Shuai Qu, Xi-Zhong Shen

**Affiliations:** 1 Department of Gastroenterology, Zhong Shan Hospital, Shanghai Medical College, Fudan University, Shanghai 200032, PR China

## Abstract

**Background:**

Methylenetetrahydrofolate reductase (MTHFR) is a key enzyme in the metabolism of folate. The non-synonymous single nucleotide polymorphism (nsSNP), C677T (Ala>Val, rs1801133), has been verified to impair enzyme activity. The association with cancer susceptibility, including hepatocellular carcinoma (HCC), has also been widely studied. The results, however, were inconsistent. To shed light on the influence of MTHFR C677T polymorphism on HCC, a meta-analysis was conducted.

**Methods:**

The meta-analysis of C677T consisted of 10 studies (1814 cases/2862 controls). The association was measured by using random-effect (RE) or fixed-effect (FE) odds ratio (OR) combined with 95% confidence intervals (CIs) according to the studies' heterogeneity.

**Results:**

Using genetic model analysis, C677T polymorphism was found to increase the risk of HCC in a complete overdominant model, which indicates that heterozygotes CT are at a lesser risk of HCC than either homozygotes CC or TT. Meta-analyses of the 10 studies showed that the TT genotype increased the risk of HCC as compared to the CT genotype: FE OR was 1.20 (95%CI: 1.00-1.45, p for heterogeneity = 0.21). When subgroup analysis was done between the HCC cases and the chronic liver disease (CLD) patients of four studies, meta-analysis showed that individuals with the TT genotype had increased HCC risk compared with those with the CT genotype: FE OR (TT vs. CT) reached 1.81 (1.22-2.71, p for heterogeneity = 0.25). Meanwhile, the C677T polymorphism also increased HCC risk in a recessive model when cases were compared to CLD patients of four studies: RE OR reached 1.85 (95%CI: 1.00-3.42, p for heterogeneity = 0.06). Overall, there was some extent heterogeneity when analyses were performed in various models. There was no publication bias.

**Conclusion:**

MTHFR C677T polymorphism increased the risk of HCC in an overdominant model, and might be a risk factor for HCC occurrence, especially in CLD patients. The association warranted further studies.

## Background

Hepatocellular carcinoma (HCC) is the fifth most common cancer in the world and the third leading cause of cancer deaths [1]. Genetic variation has been postulated to influence the variable risk for HCC observed both within and across populations. Because the liver is the main metabolizing organ and plays an important role in the detoxification or activation of carcinogens, metabolizing enzyme genes have become the best HCC candidate genes. These metabolizing enzymes include cytochrome P450 2E1 (CYP2E1), UDP-glucuronosyltransferase1A7 (UGT1A7), N-acetyltransferase (NAT2), manganese superoxide dismutase (MnSOD), and alcohol dehydrogenase-2 (ALDH2), among others [2-6].

Methylenetetrahydrofolate reductase (MTHFR) is a key enzyme in the metabolism of folate [7]. According to Entrez Gene, *MTHFR *(gene ID: 4524) maps on chromosome 1 at 1p36.3. It covers 21.20 kb, from 11789564 to 11768370 (NCBI 36, March 2006), on the reverse strand. The gene product is a 77 kD protein. Together with other enzymes, MTHFR plays a central role in folate metabolism by irreversibly catalyzing the conversion of 5,10-methylenetetrahydrofolate (5,10-methylene-THF) to 5-methyltetrahydrofolate (5-methylene-THF), the primary circulating form of folate and a cosubstrate for homocysteine methylation to methionine. In humans, folate plays a fundamental role in providing methyl groups for de novo deoxynucleotide synthesis and intracellular methylation reactions. One non-synonymous single nucleotide polymorphism (nsSNP) of MTHFR, C677T (Ala>Val, rs1801133), has been proven to impair enzyme activity [8]. From three nsSNP function prediction Web software programs, namely, SIFT http://sift.jcvi.org[9], Polyphen http://coot.embl.de/PolyPhen[10], and SNPs3D http://www.snps3D.org[11], 10 nsSNPs that possibly damage protein function were detected. The three software programs simultaneously predicted that C677T (rs1801133), which changes the amino acid Ala to Val, damages the protein function. The relationship of C677T and cancers has been widely studied. C677T was reported to be associated with prostate cancer [12], breast cancer [13], colorectal cancer [14], stomach cancer [15], bladder cancer [16], esophageal cancer [17], and leukemia [18], among others.

A number of studies have explored the correlation of MTHFR C677T polymorphism and HCC, but the results are controversial [19-26]. To shed light on the influence of MTHFR C677T polymorphism on HCC, a meta-analysis was carried out.

## Methods

### Study identification and selection

Eligible studies were identified by searching the database of PubMed, ISI Web of Knowledge, Elsevier ScienceDirect, and HuGE Navigator Web server http://www.hugenavigator.net[27] for relevant reports in English published before May 2009 using the following search terms: "MTHFR" or "methylenetetrahydrofolate reductase" and "liver cancer" or "hepatocellular carcinoma." The reports and dissection database published in the Chinese Biomedical Database (CBM), China National Knowledge Infrastructure (CNKI), and Wan Fang (Chinese) database were also searched to collect articles of case-control studies or cohort studies on associations between MTHFR polymorphisms and susceptibility to HCC before May 2009. Studies were selected if there were available data for MTHFR C677T polymorphism with the risk of HCC using a case-control or cohort design. The reference lists of retrieved articles were also reviewed to identify additional articles missed by the above search. Studies that determined the distribution of the C677T genotype in cases with HCC diagnosed by histopathological biopsy or by elevated α-fetoprotein (AFP) and distinct iconography changes (CT, MRI, and B ultrasonography), and in controls free of cancer were eligible for inclusion in the meta-analysis. Review articles, case-only articles, and repeated literatures were excluded.

### MTHFR genotyping methods

Seven articles (which included eight studies) [19-22,24-26] and one unpublished Chinese research used extra blood samples to extract genome DNA, of which seven studies used polymerase chain reaction-restriction fragment length polymorphism (PCR-RFLP) to genotype, and one article [25] (which contained two studies) used fluorogenic 5'-nuclease assay (TaqMan Assay) to genotype. One study [23] published in Chinese extracted DNA from HCC tumor tissue and peritumoral tissue for genotyping using the PCR-RFLP method.

### Data extraction and synthesis

The following information was extracted from each study: first author's surname, year of publication, ethnicity of study population, country where the study was conducted, genotyping method, and the number of cases and controls for each C677T genotype. When specific results were not directly reported, available tabular data were used to calculate them.

### Statistical methods

In order to compare the odds ratio (OR) on the same baseline, crude OR was used for the meta-analysis. Following the genetic model analysis methods suggested by Thakkinstian A et al. [28], the wild-type allele was first set as A and the variant allele as B. Then meta-analysis examined the association for the allele contrast BB vs. AA (OR1), AB vs. AA (OR2), and BB vs. AB (OR3). Next, the best genetic model was determined according to the relation of the values of OR1, OR2, and OR3. If OR1 = OR3 ≠ 1 and OR2 = 1, then a recessive model is suggested. If OR1 = OR2 ≠ 1 and OR3 = 1, then a dominant model is suggested. If OR1 = 1, OR2 = 1, and OR3 ≠ 1, then a complete overdominant model is suggested. If OR1>R2>1 and OR1>OR3>1 (or OR1<OR2<1 and OR1<OR3<1), then a codominant model is suggested. The effect of association was indicated as crude OR with the corresponding 95%CI. The pooled OR was estimated using the fixed-effect (FE) model or the random-effect (RE) (DerSimonian and Laird) model [29]. The heterogeneity between studies was tested using Q statistic [30]. If p < 0.10, the heterogeneity was considered statistically significant, and then the RE model was used. Heterogeneity was quantified using the I^2^metric, which is independent of the number of studies in the meta-analysis (I^2^<25%, no heterogeneity; I^2 ^= 25-50%, moderate heterogeneity; I^2^>50%, large or extreme heterogeneity) [31]. A cumulative meta-analysis was carried out to evaluate the trend of pooled OR. The potential publication bias was tested using the Egger regression test asymmetry and Begg's test for funnel plot. The distribution of the genotypes in the control group was tested for Hardy-Weinberg equilibrium using a goodness-of-fit chi-square test.

All analyses above were conducted using STATA, version 10.0, software (Stata Corp., College Station, Texas). All p values were two sided. A p value less than 0.05 was considered statistically significant.

The false positive report probability (FPRP), the probability of no true association between a genetic variant and disease given a statistically significant finding, depends not only on the observed p value but also on both the prior probability that the association between the genetic variant and the disease is real and the statistical power of the test. An Excel spreadsheet to calculate FPRP is included with the online material (see http://jncicancerspectrum.oupjournals.org/jnci/content/vol96/issue6) [32].

The statistical power was calculated using the PS software http://biostat.mc.vanderbilt.edu/twiki/bin/view/Main/PowerSampleSize[33,34]. Given that the gene mutation was regarded as causal, we used population-attributable risk (PAR) to refer to the proportion of disease risk in a population that can be attributed to the causal effects of the risk gene. PAR can be assessed by using the formula [35]: PAR (%) = p(OR-1)/(p(OR-1)+1) × 100%, where p is the proportion of the individuals exposed to risk gene in the general population, and OR is the pooled OR when cases and controls were compared in the risk model.

## Results

### Eligible studies

All together, eight articles (which included nine studies) published in English and one Chinese graduate paper (not in the list of references) met the inclusion criteria. One article [25] contained two different ethnic populations, so it was regarded as two studies. In total, ten studies (1814 cases/2862 controls) examined C677T polymorphism were included in the meta-analysis (Table 1).

**Table 1:** Description of the studies included in the meta-analysis of the relationship of MTHFR C677T and HCC

Author	Year	Country	Case/control	Source of controls	Cases			controls		
					CC	CT	TT	CC	CT	TT
Fabris	2009	Italy	65/236	Population and hospital	22	30	13	111	195	77
DÁmico	2009	Italy	94/308	Population and hospital	30	37	27	133	131	44
Kwak	2008	Korea	96/201	population	32	46	18	64	106	31
Yuan	2007	American	118/209	population	53	51	14	80	99	30
Yuan	2007	China	247/248	hospital	159	71	17	156	74	18
Mu	2007	China	194/391	population	50	114	30	135	199	57
Zhu	2006	China	508/543	hospital	172	226	110	173	268	102
Ventura	2005	Italy	22/162	Population and hospital	8	5	9	94	48	20
Yang	2005	China	322/185	population	229	80	13	131	46	8
Saffroy	2004	France	148/232	Population and hospital	67	69	12	92	114	26

All studies were published between 2004 and April 2009. In all studies, the cases were histologically confirmed or diagnosed by elevated AFP and distinct iconography changes (CT, MRI, and B ultrasonography). The controls were free of cancer. All ten studies reported the use of healthy controls or non-liver disease, non-cancer patients as controls. Four studies [19,24,26,36], on the other hand, included chronic liver disease (CLD) patients as controls. CLD patients were mainly liver cirrhosis (LC) patients. Liver cirrhosis was either histologically proved or diagnosed on concordant clinical, biological, and morphological criteria.

Studies were conducted in various populations of different ethnicity: Five studies were conducted in populations of Asian ethnicity [20,22,23,25] (one Chinese paper not in the list of references), and another five studies involved Europeans or Americans [19,21,24-26]. The genotype distributions of the control groups in two studies [25,26] did not conform to the HWE equilibrium (p = 0.03 and 0.001, respectively).

### Meta-analysis results

In all 10 studies, the T allele frequency of C677T was 0.37 in the control group (2106/5724) and was 0.35 in the case group (1255/3628). The T allele was more likely found in the control groups (p = 0.03). The T frequency in the European controls of five studies was 0.38 (981/2588), and that in the Asian controls of five studies was 0.36 (1125/3136). This showed that there is no inter-ethnicity difference in minor allele frequency (p = 0.11).

**Figure 1:**
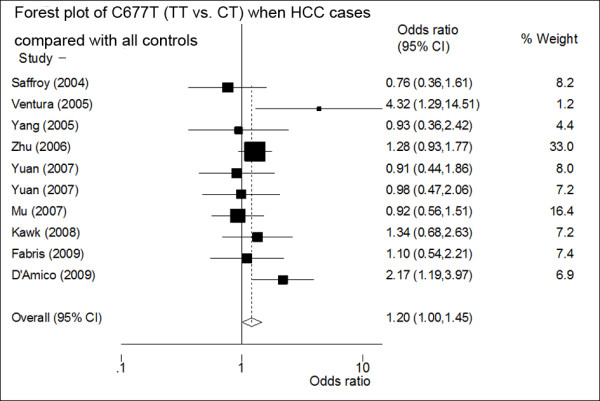
**Forest plot of the odds ratios (ORs) and 95% confidence intervals (CIs) of studies on the association between HCC and the MTHFR C677T polymorphism (TT vs. CT) of ten studies (including all controls)**. On the left, the first author of the study is followed by the publication year in parentheses. The size of the black box corresponding to each study is proportional to the sample size. The horizontal line shows the corresponding 95%CI of the OR. The combined estimate is based on a fixed-effects model shown by the diamond. The solid vertical line represents the null result.

The genetic model analysis of 10 studies (including all controls) showed pooled OR1 (TT/CC) = 1, pooled OR2 (TC/CC) = 1, and pooled OR3 (TT/CT)>1, indicating that a complete overdominant model was applicable. FE OR3 reached 1.20 (95%CI: 1.00-1.45, p for heterogeneity = 0.21) (Figure 1), showing that an overdominantly acting protective allele was applicable, that is, heterozygotes are at a lesser risk of HCC than either homozygotes (CC or TT). However, when two studies [25,26] that were not in agreement with the HWE equilibrium were excluded, the association was no longer significant (pooled OR3 = 1.18, 95%CI: 0.97-1.43, p for heterogeneity = 0.39), suggesting that the result was not stable.

The meta-analysis results under three conditions, namely, dominant model, recessive model, and allele frequency contrast (T vs. C), including 10 studies, all failed to reach statistic significance. To illustrate, RE OR reached 1.02 (95%CI: 0.85-1.23, p for heterogeneity = 0.07) for the dominant model, 1.22 (95%CI: 0.92-1.61, p for heterogeneity = 0.02) for the recessive model, and 1.08 (95%CI: 0.92-1.27, p for heterogeneity = 0.00) for the T vs. C case (Table 2). The meta-regression and sensitivity analyses found that two studies [21,26] were the main sources of heterogeneity. Therefore, heterogeneity was reduced significantly after excluding these two studies, without substantially altering the meta-analysis results.

**Table 2:** Meta-analysis results of the association of C677T polymorphisms and HCC of 10 studies.

Genetic model	OR	95%CI	p for herterogeneity	I^2^	Egger's p
TT/CC (OR1)	1.19	0.86-1.65	0.01	0.58	0.84
CT/CC (OR2)	0.96	0.84-1.10	0.42	0.02	0.71
TT/CT (OR3)	**1.20**	**1.00-1.45**	0.21	0.25	0.84
(TT+CT)/CC	1.02	0.85-1.23	0.07	0.43	0.32
TT/(CC+CT)	1.22	0.92-1.61	0.02	0.53	0.84
CT/(CC+TT)	0.92	0.81-1.05	0.65	0	0.75
T/C	1.08	0.92-1.27	0.00	0.64	0.37

**Figure 2:**
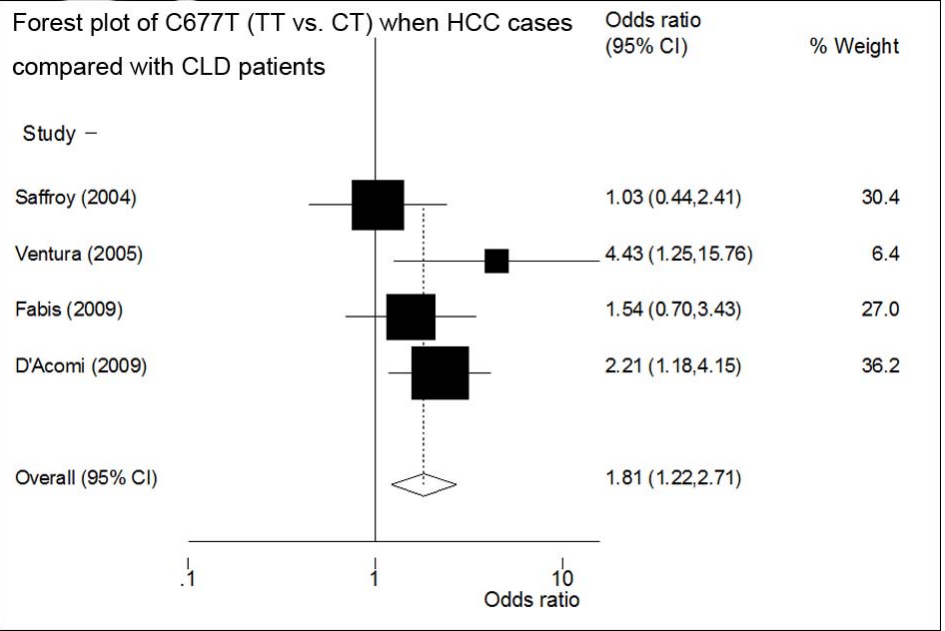
**Forest plot of the ORs and 95%CIs of studies on the association between HCC and MTHFR C677T polymorphism (TT vs. CT) when the CLD controls were compared with the HCC cases of four studies**. The size of the black box corresponding to each study is proportional to the sample size. The horizontal line shows the corresponding 95%CI of the OR. The combined estimate is based on a fixed-effects model shown by the diamond.

Stratified analyses were conducted according to the control collection (CLD patients or non-liver disease controls) and regions (Europe or Asia). The meta-analysis of four studies (including 329 HCC cases and 625 CLD patients) showed a positive association of 677TT with HCC in CLD patients: pooled FE OR3 (TT/CT) reached 1.81 (95%CI: 1.22-2.71, p for heterogeneity = 0.25) (Figure 2), indicating that a complete overdominant model was still applicable. Meanwhile, the recessive model RE OR of four studies reached 1.85 (95%CI: 1.00-3.42, p for heterogeneity = 0.06). FE OR of four studies reached 0.75 (95%CI: 0.57-0.99, p for heterogeneity = 0.92) as CT heterozygotes was compared with (CC+TT) between cases and CLD patients of four studies. Table 3 showed the results of the meta-analysis of four studies when CLD patients were compared with the cases. It is worth noting that all four studies including CLD patients as controls were from European populations, so the result was only observed from European CLD patients.

**Table 3:** Meta-analysis results of the association of C677T polymorphisms and HCC of 4 studies (including CLD controls)

Genetic model	OR	95%CI	p for herterogeneity	I^2^	Egger's p
TT/CC (OR1)	1.72	0.84-3.52	0.04	0.64	0.69
CT/CC (OR2)	0.85	0.62-1.16	0.82	0	0.44
TT/CT (OR3)	**1.81**	**1.22-2.71**	0.25	0.28	0.71
(TT+CT)/CC	1.03	0.77-1.36	0.13	0.46	0.26
TT/(CC+CT)	**1.85**	**1.00-3.42**	0.06	0.60	0.78
CT/(CC+TT)	**0.75**	**0.57-0.99**	0.92	0	0.68
T/C	1.31	0.86-2.02	0.01	0.76	0.25

Subgroup meta-analysis of (1) the comparison between the healthy controls and HCC cases of 10 studies; (2) five studies of European populations and (3) five studies of Asian populations all showed that 677T allele had a non-significantly increased risk of HCC and had a non-significant trend of overdominant model to HCC occurrence (detailed data not shown).

Publication bias analyses of 10 studies including all controls and four subgroup studies that used CLD controls both showed no publication bias: Egger's p reached 0.25-0.84 under various models.

**Figure 3:**
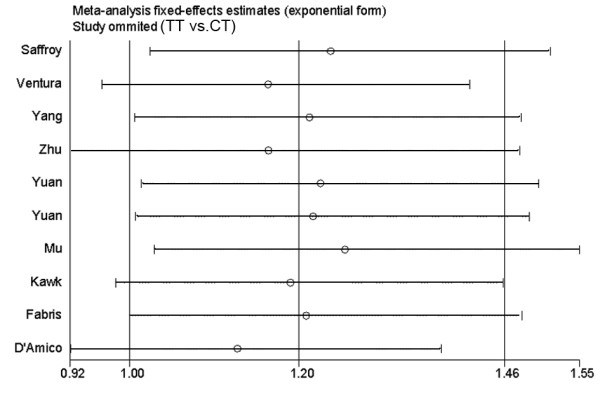
**Sensitivity analysis: examining the influence of individual studies of ten studies (TT vs. CT)**. This figure shows the influence of each study on the meta-analysis, in which the meta-analysis estimates are computed by omitting one study at a time. By default, fixed-effects analyses are displayed.

**Figure 4:**
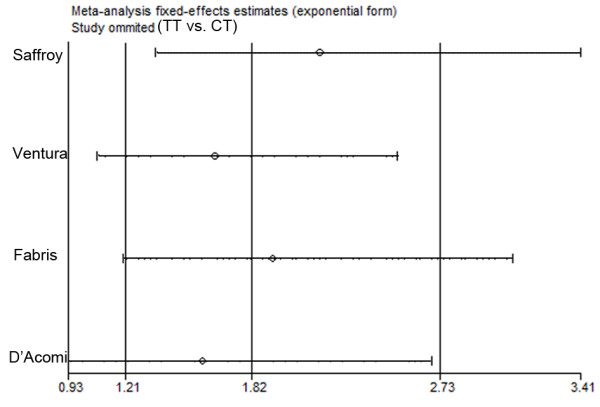
**Sensitivity analysis: examining the influence of individual studies of four studies which used CLD patients as controls (TT vs. CT)**. This figure shows the influence of each study on the meta-analysis, in which the meta-analysis estimates are computed by omitting one study at a time. By default, fixed-effects analyses are displayed.

However, the sensitivity analysis showed that the association between C677T and HCC (TT vs. CT) of 10 studies (including all cases and controls) was vulnerable: when someone studies was omitted at a time, the 95%CI of the OR3 (TT/CT) model would include 1.0 (Figure 3). Further, the sensitivity analysis showed that the association between C677T and HCC (TT vs. CT) was also vulnerable when CLD patients were used as controls (Figure 4). This is the same as the result in the recessive model (figure not shown).

When meta-analysis was performed in four studies using CLD patients as controls, the cumulative meta-analysis of the recessive model and the OR3 (TT/CT) of C677T showed a stable increased risk trend of HCC occurrence as evidences accumulated (figure not shown).

Because most HCC occurred in LC patients, MTHFR C677T polymorphism may have important clinical values. We further analyzed the reliability of the result in other ways below.

In four studies including CLD patients (mainly LC patients) as controls, there were 329 HCC cases and 625 patients. The proportion of CLD patients with TT or CC genotype was 0.53, and FE OR (TT+CC vs. CT) was 1.33. Then, the statistical power calculated by using the PS software was 0.55.

A statistically significant association (p < 0.05) can be further evaluated by estimating the false-positive report probability (FPRP) [32] at a given prior probability and statistical power. If the prior probability (incidence of HCC among LC patients) was set to 0.30, and OR = 1.33 (95%CI: 1.01-1.76) (TT+CC vs. CT), then FPRP equaled 0.18(<0.20), indicating that the association was noteworthy.

In CLD patients, PAR can be assessed by using the formula [35]: PAR (%) = p(OR-1)/(p(OR-1)+1) × 100%, where p is the proportion of the individuals exposed to risk gene (proportion of CC and TT genotype was 0.53 in the CLD patients), and OR is the pooled OR when cases and controls were compared in the risk model (OR = 1.33). Then, PAR was 14.88% (95%CI: 1.22%-28.54%).

## Discussion

HCC is a complex disease that involves multistep, multigene, and gene-environment interactions. To date, the results found on the association of MTHFR C677T polymorphism with HCC are inconsistent, and there is still no meta-analysis study that has successfully established the relationship of MTHFR C677T polymorphism and HCC.

MTHFR plays a central role in folate metabolism [7]. Individuals who are heterozygous (CT) or homozygous (TT) for MTHFR C677T polymorphism have an in vitro enzyme activity that is 65% or 30% of that of wild-type homozygous (CC) individuals, respectively [8]. Methylene-THF is involved in the conversion of deoxyuridylate monophosphate (dUMP) to deoxythymidylate monophosphate (dTMP). Low levels of 5,10-methylene-THF would lead to an increased dUMP/dTMP ratio. In addition, increased incorporation of uracil into DNA in place of thymine may increase the chance of point mutations and DNA/chromosome breakage. A less active MTHFR would lead to an accumulation of 5,10-methylene-THF; thus, a lower dUMP/dTMP ratio may reduce cancer risk [37]. Impaired MTHFR activity, on the other hand, might influence cancer risk by the level of S-adenosyl-L-methionine, the common donor of the methyl group that is necessary for maintaining the methylation patterns in DNA. The MTHFR of low activity leads to lower S-adenosyl-L-methionine levels, which consequently results in genome hypomethylation [38] and would be expected to increase the risk of some cancers [39]. Therefore, we postulated that MTHFR with a balanced or moderate activity (CT genotype) could have the best protective role on cancer occurrence.

This meta-analysis of 10 studies (1814 cases/2862 controls) showed that the CT heterozygotes of C677T polymorphism had the least risk of HCC, especially among CLD controls, supporting this hypothesis. TT homozygotes of MTHFR had the most distinct risk of HCC. The meta-analysis in the current work was the first time which used genetic analysis to explore the genetic model underlying hepatocarcinogenesis and found that MTHFR C677T polymorphism increased the risk of HCC in a complete overdominant model, suggesting it might be a risk factor for HCC, especially in CLD patients. However, sensitivity analysis showed that the association of C677T with HCC was not robust. We thought that it was mainly because of relatively small samples and low statistic power (power = 0.55). We further calculated FPRP and found it supported the positive association with HCC in LC patients (FPRP < 0.2). Population-attributable risk (PAR) is a valuable parameter to assess the influence of risk factors on disease occurrence. PAR (14.88%, 95%CI: 1.22-28.54) among the CLD patients indicated that the role of C677T polymorphism in HCC occurrence was modest.

It is worth noting that CLD patients were not used as controls in five Asian studies, and there are no such data to date. Therefore, an examination of this association in CLD patients from Asian populations is urgently needed.

Several studies have reported the association between MTHFR C677T polymorphism and various kinds of cancers, and a number of meta-analysis articles have summarized the effects [12,40-48]. According to the results, the 677T allele increased the risk of breast cancer in premenopausal women, gastric cancer, bladder cancer, esophageal cancer, multiple myeloma, and non-Hodgkin lymphoma. In contrast, it decreased the risk of prostate cancer, colorectal cancer, and acute lymphoblastic leukemia. The discrepancy of the role of C677T on different cancers has yet to be elucidated. We postulate that it is the interaction between MTHFR polymorphism and folate and the balance of 5,10-methylene-THF and 5-methylene-THF determine the susceptibility of different kinds of cancers. The carcinogenesis of breast cancer in premenopausal women, gastric cancer, bladder cancer, esophageal cancer, multiple myeloma, and non-Hodgkin lymphoma may be more related to genome hypomethylation, while the carcinogenesis of prostate cancer, colorectal cancer, and acute lymphoblastic leukemia may be more related to DNA synthesis. From the result of this meta-analysis, we postulate that both DNA synthesis and genome methylation are involved in the hepatocarcinogenesis, and genome hypomethylation may be more important. Unfortunately, few studies in this meta-analysis had provided such environment factors and made further stratified analysis impossible.

Some limitations of this meta-analysis should be acknowledged. First, the number of studies included in this article was insufficient and the sample size of individual studies was also small, leading to low statistical power. Second, there were only European studies used CLD patients as control subjects. So, the association needs to be verified in other ethnicities. Third, there were few studies have examined the gene-gene and gene-environment interactions, and so on.

## Conclusion

In conclusion, this meta-analysis found some evidences of the association between MTHFR C677T polymorphism and HCC occurrence: MTHFR C677Tpolymorphism increased the risk of HCC in a complete overdominant model, suggesting it might be a risk factor for HCC, especially in European CLD patients. The association between MTHFR C677T polymorphism and HCC warrants further examination, especially in other ethnicities. Particularly, researches including environment factors (such as folate level, alcohol, smoking habits, hepatic virus B and C, etc.) in a larger scale would be more effective.

## Abbreviations

(MTHFR): methylenetetrahydrofolate reductase; (HCC): hepatocellular carcinoma; (SNP): single nucleotide polymorphism; (OR): odds ratio; (CI): confidence interval.

## Competing interests

The authors declare that they have no competing interests.

## Authors' contributions

FJ participated in the design of the study and performed the statistical analysis. X-ZS conceived of the study, participated in its design and coordination work, and helped draft the manuscript. L-SQ helped search articles and revised the draft. All authors read and approved the final manuscript.
